# Missed nursing care and its causes and effects on moral distress in neonatal intensive care nurses

**DOI:** 10.1111/nicc.13284

**Published:** 2025-02-28

**Authors:** Burcu Bakırlıoğlu, Bengü Çetinkaya, Rabia Nur Teki

**Affiliations:** ^1^ Faculty of Health Sciences, Department of Pediatric Nursing Pamukkale University Denizli Turkey; ^2^ Servergazi State Hospital Denizli Turkey

**Keywords:** ethics, missed care, moral distress, neonatal intensive care, neonatal intensive care nurse

## Abstract

**Background:**

Deficiencies emerge in the care provided by nurses because of the complex treatment plans, shortage of labour resources and communication problems in neonatal intensive care units (NICUs). Knowing how to provide quality patient care but being unable to maintain it because of individual or institutional issues can lead to moral distress among nurses.

**Aim:**

This study aimed to examine missed nursing care in NICUs and its relationship with the moral distress experienced by nurses.

**Study Design:**

This was a cross‐sectional study. This study included a sample of 153 NICU nurses. The data were collected online between January and May 2024 using a descriptive information form, the Missed Care Survey‐Paediatric Form, and the Moral Distress Scale‐Revised for Paediatric Nurses.

**Results:**

The mean age of the participants was 31.22 years, and on average, they cared for 3.42 ± 0.96 patients per day. The medical condition of the child under care (*t* = 2.954, *p* < .001), duration of working in the NICU (*F* = 15.901, *p* < .001), inadequate number of nurses (*F* = 3.626, *p* = .029) and job satisfaction (*F* = 3.359, *p* = .037) were listed as variables affecting the reasons for missed nursing care. Communication (*β* = .275, *p* = .018) and labour resources (*β* = .216, *p* = .021), subdimensions of the Missed Care survey used in the study, were predictive factors that positively affected moral distress.

**Conclusions:**

The factors associated with missed care should be explored as strategies to reduce moral distress. Managers should focus on integrating effective communication techniques into nursing care and increasing the labour resources to reduce missed nursing care, thus improving the moral distress levels of NICU nurses.

**Relevance to Clinical Practice:**

Understanding the factors associated with missed care and developing related strategies can complement the efforts of NICU managers, educators and nurses in reducing moral distress.

**What is known about the topic**

- Neonatal nurses are unable to meet some care requirements because of individual reasons such as years of experience in the neonatal intensive care unit, level of education, burnout and lack of motivation.
- Other factors affecting missed care include equipment issues, workforce limitations or patient‐related factors.
- Neonatal intensive care continues to cause moral distress among nurses, even with advancing technology.

**What this paper adds**

- The study provides insights into the specific and relevant characteristics of nurses working in the NICU, the reasons they fail to meet care requirements and other relevant types of missed care.
- Effective communication and an increased workforce can improve the levels of moral distress among NICU nurses.
- Minimizing missed care can lead to improvements in the levels of moral distress among NICU nurses.

## 1 BACKGROUND

Nurses provide various interventions and care to improve health and manage patients' responses to diseases. Providing qualified nursing care is one of the responsibilities of nurses, who are the main elements of health care. However, planned nursing care may not be provided to patients because of certain or uncertain reasons.^1^, ^2^ Such missed interventions were first introduced in 2006 by Kalisch as ‘missed nursing care’, defined as ‘delaying or omitting the whole or a part of the planned nursing care activity’.^3^ In addition, the concept has also been described as an error of omission, which occurs when a necessary action is not performed.^4^

In neonatal care, nurses often miss health care needs such as skin‐to‐skin care, breastfeeding in the first hour after birth, creating a suitable environment, oral care for ventilated babies and educating parents about caring and oral feeding.^5^, ^6^ Reasons for missed care include individual characteristics (such as years of experience in the neonatal intensive care unit [NICU], level of education, burnout and lack of motivation), insufficient tools and equipment and communication issues among colleagues.^7^, ^8^, ^9^ Moreover, nurses caring for more than three babies are 2.5 times more likely to miss some aspects of care.^10^ NICUs are highly complex and demanding environments where a large number of multidisciplinary health care professionals provide advanced, life‐sustaining care to critically ill infants. Medical, surgical and technological advances pose new challenges in NICUs, such as coordinating team‐based care and ethical challenges related to end‐of‐life care (Epstein & Brill).^11^ Providing care for patients who have the risk of not surviving, poorly organized end‐of‐life care, intensive working conditions without support for nurses and providing care without participation in the decision‐making process are associated with moral distress.^12^ In addition, factors such as futile and end‐of‐life care, conflicts with physicians, nurse performance and authority, poor teamwork, decision‐making regarding treatment processes and patient care, limited human resources and equipment, medical errors, patient restraints, burnout and nurses' age and work experience may contribute to the high prevalence of moral distress in intensive care.^13^, ^14^, ^15^

Moral distress refers to negative stress symptoms nurses experience when their actions conflict with their ethical values, principles and responsibilities during ethical dilemmas.^16^ Disagreements over end‐of‐life care, medical benefits and futility and complex care plans are some of the most common causes of moral distress in NICUs.^17^ Additionally, NICU nurses report difficulties in patient care because of increased workloads, poverty of family members and parental interference influenced by religious beliefs, all of which can contribute to their moral distress.^18^ In addition, neonatal intensive care nurses who experience moral distress are highly likely to leave their current positions, and some nurses leave their jobs because of moral distress.^19^ The desire to leave their current positions and a changing approach to patient care are among the factors that are significantly associated with nurses' moral distress.^20^ Begjani et al.^21^ found that factors such as decreased quality of care and perception of futile care were directly associated with moral distress. Although moral distress is directly related to burnout resulting from excessive and prolonged stress, it can also lead to other conditions that contribute to burnout, such as feelings of helplessness, lack of self‐confidence, anxiety and disappointment. It is additionally associated with missed nursing care and job burnout related to personal accomplishment.^14^, ^22^ Strategies to reduce moral distress and resulting burnout in NICU nurses should focus on addressing issues of futile care, compromised care, untruthful care and missed care.^22^, ^23^ As a result of moral distress, NICU nurses may experience anger, sadness, loss of appetite, weight loss, headaches,^18^ frustration, disappointment and a sense of powerlessness.^24^

A literature review revealed no studies examining the relationship between moral distress and missed nursing care among NICU nurses. When NICU nurses miss nursing care for various reasons, they act contrary to the professional ethical principles they were taught, resulting in moral distress. Therefore, this study aimed to examine the individual factors influencing missed nursing care and moral distress and guide the development of strategies to reduce moral distress among NICU nurses.

## 2 METHODS

### 2.1 Study design and sample

This was a cross‐sectional study conducted in NICUs, and its population consisted of all NICU nurses in Turkey. The sample size was calculated using the G*Power program (Heinrich‐Heine‐Universität Düsseldorf; version 3.1.9.7).^25^ The study sample needed to include at least 147 nurses for an effect size value (*d*) of 0.3,^26^ *α* level of 5% and 95% power (1 − *β*) level. A total of 153 NICU nurses who met the criteria and volunteered to participate were included in the study.

### 2.2 Data collection

The data for this study were collected through social media and online communication groups via a survey created using Google Forms. Before starting the survey, participants were informed about the aim and significance of the study, assured that their information would remain confidential and used solely for this study and provided with the contact details of the researcher. The completion of the survey took approximately 10 min. The study included all nurses who agreed to participate, had been working in the NICU for at least 6 months and were reachable online.

### 2.3 Data collection tools

#### 2.3.1 Descriptive information form

This form was developed by the researchers based on a thorough review of the relevant literature. It consists of eight questions regarding participants' educational level, years of experience in intensive care, frequently worked shifts, average weekly overtime hours, job satisfaction, adequacy of nurse staffing, quality of care and participation in certification programs.

#### 2.3.2 Missed Care Survey‐paediatric form

The Missed Care (MISSCARE) survey was developed by Bagnasco et al.,^27^ and its Turkish adaptation was conducted by Calıkusu et al. (2020). The survey consists of two parts: Section A and Section B. Section A includes 29 items listing nursing activities, which are responded to on a 5‐point Likert‐type scale (always = 5; never and not applicable = 1). This section assesses the frequency of missed nursing care during nurses' most recent shifts. Section B includes 16 items about possible reasons for missed nursing care, answered on a 4‐point Likert‐type scale (important = 4; not important = 1). In this section, the mean score of the three subdimensions (labour resources, communication and material resources) related to the reasons for missed care is calculated. In the Turkish adaptation of the scale, the reliability coefficients for the subdimensions of labour resources, communication and material resources, and the entire survey were 0.82, 0.87, 0.88 and 0.9, respectively (Calıkusu et al., 2020). In the present study, the reliability coefficient was 0.73 for labour resources, 0.85 for communication, 0.82 for material resources and 0.90 for the entire survey. In Section A of this survey, the responses to each item are calculated as both frequency (*n*) and percentage (%), while the average score is calculated in Section B. There is no cut‐off point for the total score in Section B; however, an increase in the average score indicates a rise in the reasons for missed nursing care.

#### 2.3.3 Moral Distress Scale‐Revised for paediatric nurses (MDS‐R)

The MDS‐R, developed by Hamric et al.,^28^ is a Likert‐type scale used to assess the moral distress levels of paediatric nurses. The Turkish validity and reliability study of the scale was conducted by Kovancı.^29^ The MDS‐R includes five subdimensions: (1) providing benefit—do no harm, (2) autonomy of the patient and family, (3) professional autonomy, (4) administrative ethics and (5) patient advocacy. The scale measures discomfort (0: Never, 1: Low, 2: Moderate, 3: Intense, 4: Very Intense) and frequency (0: Never, 1: Less Frequently, 2: Occasionally, 3: Frequently, 4: Very Frequently) on a 5‐point Likert‐type scale in two columns. The MDS‐R was developed to measure both the frequency and intensity of moral distress experienced by paediatric nurses. The overall moral distress score was obtained by multiplying the frequency and discomfort scores, with total scores ranging from 0 to 336. The scale does not have a cutoff point, and higher scores indicate higher levels of moral distress. In the Turkish adaptation, the reliability coefficient was 0.86, and in this study, it was 0.89.

### 2.4 Compliance with ethical standards

This study was approved by the Non‐Interventional Clinical Studies Ethics Committee (approval number: 208264, date: 07.03.2024). Informed consent was obtained from all the participants before starting the study. The entire study was conducted following the ethical standards of the research committee and the Declaration of Helsinki.

### 2.5 Data analysis

The data were analysed using the IBM SPSS statistics, version 25. Continuous variables were expressed as mean ± standard deviation and categorical variables as percentage and frequency. Skewness and kurtosis values were used as reference to determine whether the analysed data had a normal distribution. The independent‐sample *t* test, one‐way analysis of variance and Mann–Whitney *U* test were used to analyse the relationship between the mean scores of both scales and sociodemographic characteristics. The correlation between the subdimensions of the two scales was examined using correlation and regression analyses to investigate the predictors of the MISSCARE‐B. A *p‐*value <.05 indicated a statistically significant difference.

## 3 RESULTS

A total of 153 nurses working in the NICU were included in the study. The mean age of the participants was 31.22 ± 7.6 years (range: 23–53 years), and all of them were female. The average number of patients cared for by the nurses in 1 day was 3.42 ± 0.96 (range: 2–7). The descriptive information of participants is presented in Table 1.

**TABLE 1:** Distribution of the descriptive characteristics of nurses.

Descriptive information	*n*	%
Education level		
High school	18	11.8
Graduate	99	64.7
Postgraduate	36	23.5
Duration of work in the NICU		
6 months to 5 years	93	60.8
5–10 years	25	16.3
10 years and more	35	22.9
Shift mostly worked		
08:00–16:00	40	26.1
16:00–08:00	81	52.9
08:00–08:00	32	20.9
Average overtime hours (per month)		
0–24 h	58	37.9
24–48 h	52	34.0
48 h and more	43	28.1
Job satisfaction		
Satisfied	101	66.0
Not satisfied	20	13.1
Undecided	32	20.9
Does the medical condition and care procedure of the child you care for affect the quality of your care?		
Yes	109	71.2
No	44	28.8
Do an adequate number of nurses work in your unit?		
Yes	32	20.9
No	97	63.4
Undecided	24	15.7
Have you participated in any certification program for neonatal care (e.g. NRP training)?		
Yes	93	60.8
No	60	39.2

Abbreviation: NRP, The Neonatal Resuscitation Program®.

The top three nursing practices that the participants missed the most and least on the MISSCARE‐A form are presented in Table 2.

**TABLE 2:** MISSCARE‐A form.

Nursing interventions	Never		Rarely		Sometimes		Often		Always	
	*n*	%	*n*	%	*n*	%	*n*	%	*n*	%
Top three most frequently missed nursing practices										
Response to call light, intervention request, or alarm initiated in 5 min (i.e. monitor, infusion pumps and ventilator)	38	24.8	12	7.8	9	5.9	38	24.8	56	36.6
Central line and peripheral line site care per protocol	42	27.5	14	9.2	15	9.8	34	22.2	48	31.4
Satisfaction of eating needs according to child's clinical conditions (i.e. encouraging oral feeding and/or nutrition at the request of the newborn; encouraging a correct alimentation according to the personal taste)	34	22.2	44	28.8	8	5.2	27	17.6	40	26.1
Top three least frequently missed nursing practices										
Hand washing	73	47.7	58	37.9	2	1.3	2	1.3	18	11.8
Attendance at daily rounds at the bedside	60	39.2	20	13.1	10	6.5	46	30.1	17	11.1
Monitoring intake/output of solid and liquid food	52	34.0	39	25.5	6	3.9	20	13.1	36	23.5

Abbreviation: MISSCARE, Missed Care.

Nurses reporting that the medical condition of the child they cared for affected their quality of care had statistically significantly higher mean total MDS‐R (*t* = 0.175, *p* = .035) and MISSCARE‐B (*t* = 2.954, *p* = *p* < .001) scores than those who reported that it did not. In addition, the nurses who had worked in the NICU for 6 months to 5 years had higher mean total MISSCARE‐B scores than those who had worked longer (*F* = 15.901, *p* ≤ .001). Nurses who found the number of nurses working in their unit insufficient had higher scores compared with those who found it sufficient (*F* = 3.626, *p* = .029). Those who said ‘I am dissatisfied’ and ‘I am undecided about my job’ also had higher scores compared with those who were satisfied (*F* = 3.359, *p* = .037) (Table 3).

**TABLE 3:** The association between the descriptive information of NICU nurses on the mean scores of MDS‐R and MISSCARE‐B.

	MDS‐R	MISSCARE‐B
Education level		
High school	77.44 ± 43.63	2.79 ± 0.37
Graduate	80.73 ± 50.25	2.85 ± 0.60
Postgraduate	69.33 ± 37.12	2.89 ± 0.59
	*F* = 0.785, *p* = .458	*F* = 0.172 *p* = .842
Duration of work in the NICU		
6 months to 5 years^a^	83.79 ± 47.36	3.04 ± 0.49
5–10 years^b^	66 ± 48.19	2.43 ± 0.45
10 years or more^c^	69.71 ± 42.08	2.65 ± 0.69
	*F* = 2.119, *p* = .124	*F* = 15.901 *p* < .001 *a* > *b,* *p* < .001 *a* > *c,* *p* < .001
Shift mostly worked		
08.00–16.00	71.20 ± 42.84	2.88 ± 0.65
16.00–08.00	80.13 ± 4630	2.82 ± 0.58
08.00–08.00	79.50 ± 52.71	2.91 ± 0.52
	*F* = 0.518, *p* = .59	*F* = 0.320, *p* = .726
Average overtime hours		
0–24 h^a^	72.24 ± 45.61	2.65 ± 0.58
24–48 h^b^	79.07 ± 41.84	2.94 ± 0.62
48 h and more^c^	83.27 ± 53.55	3.02 ± 0.47
	*F* = 0.723, *p* = .487	*F* = 6.056*, p* = .003 *b* > *a*, *p* = .025 *c* > *a*, *p* = .005
Job satisfaction		
I am satisfied^a^	75.75 ± 47.48	2.77 ± 0.63
I am not satisfied^b^	94.20 ± 48.32	3.01 ± 0.32
I am undecided^c^	73.37 ± 42.19	3.03 ± 0.52
	*F* = 1.483, *p* = .23	*F* = 3.359, *p* = .037 *b* > *a*, *p* = .013 *c* > *a*, *p* = .022
Does the medical condition and care procedure of the child you care for affect the quality of your care?		
Yes	82.94 ± 44.93	2.97 ± 0.57
No	64.59 ± 48.91	2.57 ± 0.63
	*t* = 0.175, *p* = .035	*t* = 2.954, *p* < .001
Do an adequate number of nurses work in your unit?		
Yes^a^	66.75 ± 41.31	2.62 ± 0.62
No^b^	78.31 ± 46.73	2.94 ± 0.56
Undecided^c^	89.58 ± 51.78	2.83 ± 0.57
	*F* = 1.680, *p* = .190	*F =* 3.626, *p =* .029 *b* > *a*, *p* = .022
Have you participated in any certification program for neonatal care?		
Yes	81.26	72.94
No	70.40	83.30
	*U* = 2394, *p* = .139	*U =* 2412, *p* = .157

*Note*: *F:* One‐way analysis of variance, *t*: independent‐samples *t* test, *U* = Mann–Whitney *U* test.

Abbreviations: MDS‐R, Moral Distress Scale‐Revised for Paediatric Nurses; MISSCARE, Missed Care; NICU, neonatal intensive care unit.

The results of the multiple linear regression analysis regarding the prediction of moral distress among NICU nurses using the subdimensions of MISSCARE are presented in Table 4. This analysis showed that communication (*β* = .275, *p* = .018) and labour resources (*β* = .216, *p* = .021) positively affected moral distress. Both communication (*β* = .343, *p* = .003) and labour resources (*β* = .217, *p* = .020) were also positive predictors of moral distress frequency. However, the reasons for missed nursing care were not significant predictors of moral distress. Overall, the reasons for missed nursing care explained 17% of the change in moral distress score (*R* ^2^ = 0.179), 11% of the change in moral distress discomfort levels (*R* ^2^ = 0.112) and 18% of the change in moral distress frequency (*R* ^2^ = 0.182).

**TABLE 4:** Predictors of moral distress for neonatal nurses.

	MDS‐R total							MDS‐R intensity							MDS‐R frequency						
	*B*	SE	*β*	*t*	*p*	95% CI		*B*	SE	*β*	*t*	*p*	95% CI		*B*	SE	*β*	*t*	*p*	95% CI	
						LLCI	ULCI						LLCI	ULCI						LLCI	ULCI
Constant	−22.688	19.009		−1.194	.235	−60 251	14 875	15.214	6.386		2.382	.018	2595	27 833	3.013	5.431		0.555	.580	−7719	13 746
Material resources	−1.285	6.667	−0.021	−0.193	.847	−14 459	11 889	1.886	2.240	0.094	0.842	.401	−2540	6311	−2.092	1.905	−0.118	−1.098	.274	−5856	1672
Communication	18.324	7.628	0.275	2.402	.018	3250	33 397	3.675	2.563	0.170	1.434	.154	−1389	8739	6.558	2.180	0.343	3.009	.003	2251	10 865
Labour resources	16.771	7.186	0.216	2.334	.021	2572	30 970	3.103	2.414	0.124	1.285	.201	−1667	7873	4.821	2.053	0.217	2.348	.02	,764	8878
Model summary	*F* = 10.826, model (*p*) < .001, *R* ^2^ = 0.179							*F* = 6.281, model (*p*) < .001, *R* ^2^ = 0.112							*F* = 11.062, model (*p*) < .001, *R* ^2^ = 0.182						

Abbreviations: CI, confidence interval; MDS‐R, Moral Distress Scale‐Revised for Paediatric Nurses.

## 4 DISCUSSION

### 4.1 Possible reasons for missed care

Studies on missed nursing care involving paediatric patients often show significant differences both among themselves and from the findings of this study. For instance, the most frequently missed care identified among NICU nurses was providing developmental care to infants,^30^ whereas another study found that the care of central and venous catheters was most frequently missed.^10^ According to Arıkan and Esenay,^31^ gastrostomy care was the most missed care among paediatric emergency nurses; however, Elmaoğlu and Özdemir^7^ who conducted a study involving all paediatric nurses identified lifting and walking the child as a significant missed care. The differences among these findings may be attributed to patient diversity, individual differences among nurses or varying working dynamics. The results of this study showed that the most frequently missed nursing care was ‘response to call light, intervention request, or alarm initiated in 5 min (e.g. monitor, infusion pumps, ventilator)’, a finding similar to the findings of Daraghmeh et al.^32^ with nurses working in the NICU. This missed care may be because of the extreme sensitivity of the devices monitoring vital signs; even normal newborn movements can trigger alarms, leading nurses to prioritize other situations they perceive as more urgent. The failure in ‘central line site and peripheral line site care per protocol’ ranked second in missed nursing care. This may be attributed to the fact that nurses do not use the guidelines for evidence‐based practices in intravenous catheter care.^33^ In addition, missed nursing care significantly mediates the development of catheter‐related venous phlebitis.^34^ This increases the mortality and length of stay in the NICU. Failure in ‘satisfaction of eating needs according to child's clinical conditions (i.e., encouraging oral feeding and/or nutrition at the request of the newborn; encouraging a correct alimentation according to the personal taste)’ ranked third in missed nursing care. This missed care may be related to the clinical condition of the baby (e.g. orogastric catheter, parenteral nutrition) rather than the nurse. In general, the high workload in the NICU (e.g. the nurse caring for a large number of patients) may be the reason for the first three missed nursing care situations identified in this study. As a result, tasks may be prioritized and completed based on their perceived importance and urgency. In this case, care that nurses think is not critically important or does not require an urgent intervention is often missed.

The three least frequently missed nursing care activities reported in this study were hand washing, attendance at daily rounds at the bedside and monitoring intake/output of solid and liquid food. These findings were consistent with those of Calikusu Incekar et al.,^35^ Bartoníčková et al.^36^ and Elmalıoğlu and Özdemir,^7^ Increasing demands on the nursing workforce have limited the amount of time nurses can allocate to each patient under their care. Therefore, care that does not require much labour and time and the essentials of nursing care engrained in all nurses is often performed without being missed.

### 4.2 Factors affecting missed care and moral distress

This study found that the medical condition of the child being cared for and the care process affected moral distress among nurses. The perception of disproportionate care because of the use of technology that is not in the best interest of the patient and the inability of health care professionals to advocate for the child are the main apparent causes of moral distress; however, other underlying factors can also affect moral distress (Prentice et al., 2016).^15^ The shorter working periods in the NICU, high average overtime hours, dissatisfaction with the work, inadequate number of nurses and perception that the child's medical condition affects the quality of care are factors that led to an increase in the mean MISSCARE‐B score. Missed care is more likely to be common in units where nurses work longer hours, and long working hours may increase the risk of compromised care and reduce the quality of care (Senek et al^37^.; Kohanová et al.^38^). Consistent with the present study, Mainz et al.^39^ found that inadequate number of nurses and the patient's medical condition were the causes of missed nursing care and that nurses with less than 5 years of work experience had more instances of missed care.^39^ However, the increase in missed care among nurses with more than 5 years of work experience in the present study may be because of increased burnout levels resulting from the work and emotional burden in the NICU.

### 4.3 Predictors of moral distress

The mean MDS‐R (paediatric) score of the nurses participating in the study was 77.18 ± 46.92 (low moral distress). One of the most important findings of this study was that communication and labour resources were the predictors of both the moral distress experienced by nurses and the frequency of moral distress. No previous study examining the relationship between the causes of missed nursing care and moral distress in NICU nurses was found in the literature review. A study conducted with a large sample identified five main causes of moral distress among health care professionals working in NICUs: infant‐centred reasons (severity of the disease, predicted outcomes and disproportionate care), management plans, family‐centred reasons, parental decision‐making process and health care professional–related factors. The study also found that nurses experienced more moral distress compared with neonatologists.^40^ A qualitative study conducted with nurses working with adult patients in different units reported that missed nursing care caused moral distress. The study concluded that nurses experienced moral distress when engaged in activities that conflicted with the professional ethical principles they had been taught, including autonomy, beneficence, non‐maleficence, justice, privacy and confidentiality.^41^ Therefore, nurses who cannot fulfil patient care requirements in the NICU because of workforce or communication‐related issues may act against the professional ethical principles they were taught, leading to moral distress.

The reasons for missed nursing care were predictors of intensity and the frequency of moral distress; however, they did not affect the discomfort level of moral distress. Epstein and Hamric^42^ suggested that moral distress for an individual would increase with repeated exposure to distress (‘crescendo effect’). Accordingly, the reasons for missed nursing care may increase the frequency and level of moral distress. Considering communication‐related problems, organizing training programs on effective communication methods and increasing teamwork among nurses can reduce moral distress.^43^, ^44^ In this regard, the in‐service training programs for nurses, both in their undergraduate education and for those currently working, should focus especially on effective communication skills. The task distribution that increases teamwork skills among nurses can also help address communication‐related problems. Therefore, nurse managers and educators are advised to implement such task assignments. Moreover, the perspectives of all members of the health care team and all stakeholders, including parents, should be mutually understood to reduce the moral distress experienced by nurses.^15^ In addition, multidisciplinary team communication should be shaped using an effective, transparent, supportive and solution‐oriented approach to reduce moral distress among nurses. Strengthening intra‐team communication not only reduces moral distress but also enhances nurses' job satisfaction and fosters team cohesion. In this context, communication should be considered not only as a means of problem‐solving but also as a tool for building connections among team members and working for a common goal.

An unexpected increase in patient volume and/or acuity, worsening patient conditions, insufficient nursing staff and inadequate number of nursing assistants were identified as workforce‐related causes of missed care.^27^, ^35^ A study conducted in the NICU found that the lack of nurses, increased number of admissions or discharges, increase in the number of patients, emergencies or deterioration of patient conditions and various interruptions during work were the main causes of missed care.^30^ These labour‐related issues may lead to incomplete care because of disruption in the workflow, resulting in moral distress. Another strategy to reduce moral distress among NICU nurses is to address labour‐related problems. The number of nurses per patient is still insufficient in Turkey, making it necessary to employ a sufficient number of nurses in all units, especially in NICUs. In Turkey, the number of nurses/midwives per 100 000 people is determined as 356 in official procedural data. No national data have been found on the newborn:nurse ratio. However, a large‐scale study conducted in recent years with NICU nurses (*n* = 2917) reported that 4.22 ± 2.95 patients were cared for during the day shift and 6.26 ± 5.13 during the night shift.^45^, ^46^ Reducing the burden on nurses caring for patients expected to require increased care, resolving issues with team cooperation in emergencies or acute situations and increasing the number of assistant personnel may play an important role in reducing the frequency and intensity of moral distress.

## 5 LIMITATIONS

The study data were collected using a survey created with Google Forms. Hence, the limitations of this study were the factors affecting the sincerity of NICU nurses in responding to the research questions and their inability to fully express factors affecting their emotional states toward moral distress.

## 6 IMPLICATIONS FOR PRACTICE

Neonatal nurses miss some of the nursing care because of individual and environmental factors grouped into communication, labour resources and material resources. These factors also make nurses encounter situations incompatible with their ethical values, resulting in moral distress. Neonatal nurses miss some of the nursing care because of individual and environmental factors grouped into communication, labour resources and material resources. These factors also make nurses encounter situations incompatible with their ethical values, resulting in moral distress. Managing nurses' workloads and resources, analysing nursing work processes, supporting nurses with longer career spans, reducing overtime hours and increasing job satisfaction are potential interventions to prevent missed care. In addition, understanding the factors associated with missed care and developing related strategies can complement the efforts of NICU managers, educators and nurses in reducing moral distress. All members of the health care team and parents need to have a mutual understanding of the perspectives of all stakeholders to improve the ethical environment and ultimately reduce moral distress. Nurses may face situations incompatible with their ethical values because of insufficient resources, high workloads and complex patient conditions. These may lead to various negative consequences, especially patient safety, both personally and professionally. Therefore, minimizing missed care and providing a supportive work environment are crucial to reducing moral distress among nurses. Managing nurses' workloads and resources, analysing nursing work processes, supporting nurses with longer career spans, reducing overtime hours and increasing job satisfaction are potential interventions to prevent missed care. In addition, understanding the factors associated with missed care and developing related strategies can complement the efforts of NICU managers, educators and nurses in reducing moral distress. All members of the health care team and parents need to have a mutual understanding of the perspectives of all stakeholders to improve the ethical environment and ultimately reduce moral distress. Nurses may face situations incompatible with their ethical values because of insufficient resources, high workloads and complex patient conditions.

## 7 CONCLUSIONS

This study examined the factors associated with missed nursing care among NICU nurses and their impact on moral distress. It was found that nurses frequently fail to deliver certain aspects of nursing care, which contributed to increased moral distress. To provide the highest quality nursing care for neonates, it is essential to minimize both missed care and moral distress. In this context, further detailed and in‐depth research focusing on NICU nurses is required. Such studies may provide a foundation for the development of strategies by nursing authorities to enhance care quality.

Educational interventions aiming to increase nurses' awareness about missed care and equip them with the skills to analyse and prevent such occurrences should be implemented. Incorporating effective communication techniques into in‐service training programs can strengthen both intragroup and intergroup communications, thereby contributing to the prevention of moral distress. Additionally, fostering teamwork and establishing psychological support mechanisms are recommended. Ensuring adequate nurse staffing levels in NICUs is regarded as a fundamental requirement for delivering high‐quality patient care. Furthermore, strategies aiming to improve workforce resources such as increasing staff numbers, reducing overtime hours and balancing workload are critical for mitigating the moral distress experienced by NICU nurses.

## ETHICS STATEMENT

For this study, a research permit was obtained from the non‐interventional clinical research ethics committee of Pamukkale University with the permit no. 208264, 07.03.2024. *Consent was obtained from all participants who accepted the research.

## Data Availability

The data that support the findings of this study are available from the corresponding author upon reasonable request.
